# Effect of Visceral Adipose Tissue on Major Depressive Disorder: A Mendelian Randomisation Research

**DOI:** 10.62641/aep.v53i5.1972

**Published:** 2025-10-05

**Authors:** Xin Li, Xiaoling Zhou, Yang Li, Chen Lei

**Affiliations:** ^1^Department of Nutrition, General Hospital of Ningxia Medical University, 750004 Yinchuan, Ningxia, China; ^2^Department of Nephrology, General Hospital of Ningxia Medical University, 750004 Yinchuan, Ningxia, China; ^3^Department of Geriatrics and Special Needs, General Hospital of Ningxia Medical University, 750004 Yinchuan, Ningxia, China

**Keywords:** visceral adipose tissue, major depressive disorder, mendelian randomisation analysis, genetic predisposition to disease

## Abstract

**Aims:**

Visceral adipose tissue (VAT) is associated with major depressive disorder (MDD) in observational studies, but these findings are susceptible to confounding and reverse causation. This study employed a two-sample Mendelian randomisation (MR) approach to assess the causal relationship between VAT and MDD.

**Methods:**

We selected 221 single nucleotide polymorphisms associated with VAT mass in 325,153 individuals of European ancestry from UK Biobank as instrumental variables. Summary-level genetic data for MDD (59,851 cases and 113,154 controls) were accessible from the Psychiatric Genomics Consortium database. Primary MR analysis used the inverse-variance weighted (IVW) method, with weighted median and MR-Egger approaches as sensitivity analyses. Additional tests, including MR-Pleiotropy RESidual Sum and Outlier (PRESSO) and leave-one-out analysis, were conducted to evaluate pleiotropy and robustness.

**Results:**

Genetically predicted higher VAT was significantly associated with increased MDD risk (odds ratio (OR) 1.179, 95% confidence interval (CI) 1.082–1.285, *p* < 0.001) based on IVW analysis. Sensitivity analyses yielded consistent results (weighted median OR 1.269, 95% CI 1.139–1.414, *p* < 0.001; MR-Egger OR 1.330, 95% CI 1.023–1.728, *p* = 0.034). Heterogeneity was observed (Cochran’s Q = 353.14, *p* < 0.001), with no evidence of horizontal pleiotropy (MR-Egger intercept *p* = 0.342).

**Conclusion:**

Our findings supported a causal relationship between increased VAT mass and elevated MDD risk. These results suggested that reducing VAT may be a potential strategy for preventing or mitigating MDD.

## Introduction

Major depressive disorder (MDD) is a severe psychiatric condition characterised by persistent low mood, anhedonia, cognitive impairment and somatic symptoms such as sleep disturbances or appetite changes [1]. Extensive longitudinal studies have consistently indicated that MDD is associated with an increased risk of various physical illnesses, including diabetes mellitus, cardiovascular diseases, stroke, hypertension, obesity, cancer, cognitive decline and Alzheimer’s disease [2]. The aetiology of MDD is multi-factorial, involving alterations in psychoneuroimmunoendocrinological pathways, metabolic dysregulation, oxidative stress and disruption of the microbiota–gut–brain axis [3]. The heritability of MDD is estimated to be around 35%, indicating that environmental and biological components contribute to disease development [4].

Among the emerging contributors to MDD pathophysiology, visceral adipose tissue (VAT) has garnered increasing attention. VAT, a type of fat stored in the abdominal cavity, is metabolically active and participates in various physiological and pathological processes [5, 6]. Besides acting as a passive storage site for excess calories, VAT functions as an active endocrine organ that secretes several bioactive molecules, including inflammatory cytokines, adipokines and hormones [6, 7, 8]. Recent studies have highlighted the role of VAT in neuroinflammation and cognitive dysfunction through mechanisms involving pro-inflammatory cytokines such as interleukin-1 beta (IL-1β) and tumour necrosis factor-alpha (TNF-α) [7, 8, 9, 10]. VAT-derived inflammatory signals can directly influence brain function by activating immune pathways, including microglial IL-1 receptor type 1 (IL-1R1) signalling in the hippocampus, which is associated with cognitive impairment [7, 8]. Furthermore, emerging evidence highlights the interplay among visceral adiposity, systemic inflammation and hippocampal dysfunction, with cytokines such as TNF-α contributing to synaptic deficits and neurovascular dysregulation [9]. These findings suggested that VAT may promote depressive phenotypes through inflammatory damage to brain circuits that regulate mood and cognition.

In addition to inflammatory signalling, VAT influences insulin resistance, hypothalamic–pituitary–adrenal (HPA) axis activation and adipokine imbalance (e.g., leptin resistance and reduced adiponectin), all of which are established features of MDD pathophysiology [11, 12, 13, 14]. Taken together, VAT may serve not only as a marker of metabolic risk but also as an active mediator of psychiatric vulnerability.

Although VAT and general obesity, typically measured by body mass index (BMI), are both related to adiposity, they differ in biological effects. VAT is closely linked to systemic inflammation and metabolic dysregulation, whereas BMI is a general proxy for body size that does not differentiate fat distribution [15]. Thus, VAT may exert a unique effect on MDD risk independent of BMI. Mendelian randomisation (MR) offers a valuable framework to assess this relationship by utilising genetic variants associated with VAT mass as instrumental variables (IVs) [16]. Although some overlap with BMI-related variations may exist, careful single nucleotide polymorphism (SNP) selection and sensitivity testing can help minimise bias due to pleiotropy [16, 17].

Given the limitations of observational studies in establishing causality, particularly due to confounding and reverse causation, MR offers a quasi-experimental approach that mimics randomised controlled trials [18]. In this study, we applied a two-sample MR design to investigate whether increased VAT mass causally contributes to the risk of MDD. On the basis of prior biological and genetic evidence, we hypothesise that high VAT mass is associated with an increased risk of MDD.

## Methods

### Study Design

**Fig. 1.**
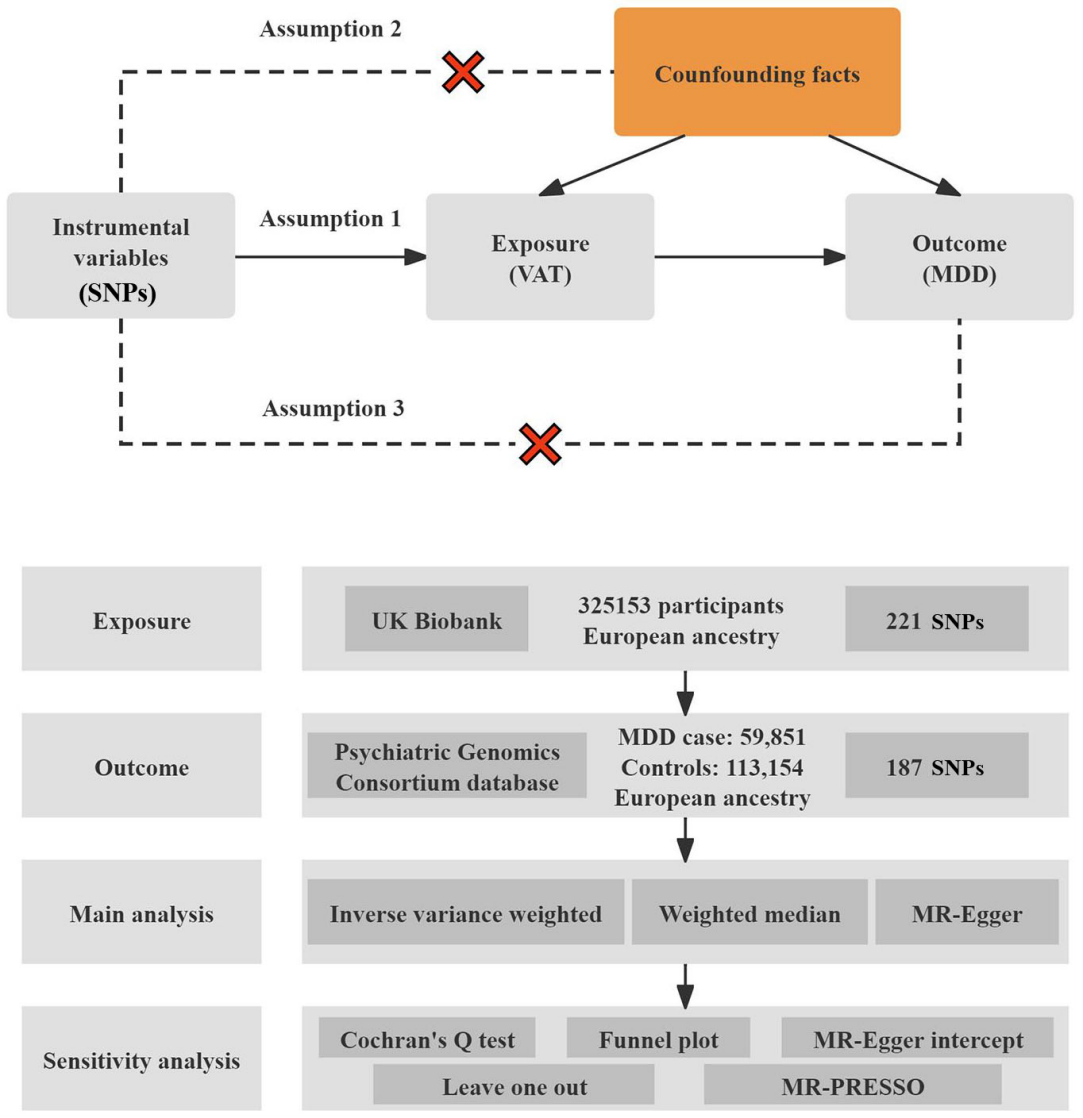
**Overview of the study design and its three core assumptions**. Three key assumptions formed the basis of this article: (1) A strong connection was established between IVs and the exposure (VAT). (2) IVs should remain free from influence by any confounding factors. (3) IVs should influence the outcome (MDD) solely via the exposure (VAT), with no alternative pathways involved. VAT, visceral adipose tissue; MDD, major depressive disorder; SNPs, single nucleotide polymorphisms; MR, Mendelian randomisation; PRESSO, Pleiotropy RESidual Sum and Outlier; IVs, instrumental variables.

The study design flowchart is shown in Fig. 1. We employed a two-sample MR framework to explore the potential causal relationship between VAT and MDD, using SNPs strongly associated with VAT as IVs. MR studies are based on three key assumptions: (1) the genetic variants are significantly associated with the exposure (VAT); (2) the variants are independent of confounding factors; and (3) the variants influence the outcomes (MDD) only through the exposure, without horizontal pleiotropy (VAT) [19].

### Data Source

Genetic instruments for VAT were obtained from a large genome-wide association study (GWAS) by Karlsson *et al*. [16], which included 325,153 individuals of European ancestry from the UK Biobank. Summary-level data for VAT were retrieved from the GWAS Catalog (https://www.ebi.ac.uk/gwas/downloads/summary-statistics). SNPs significantly associated with VAT (*p* < 5 × 10^-8^) were filtered using the ‘subset’ function in R (version 4.3.3; R Foundation for Statistical Computing, Vienna, Austria). To ensure independence, we removed SNPs in linkage disequilibrium using the ‘clump_data’ function from the ‘Two-Sample MR’ package (version 0.5.11; University of Bristol, Bristol, UK). SNPs with F-statistics >10 were retained, yielding 221 genetic instruments (**Supplementary Table 1**).

Outcome data for MDD were obtained from the Psychiatric Genomics Consortium, based on a European ancestry cohort consisting of 135,458 MDD cases and 344,901 controls [20]. For this MR analysis, we used a subset comprising 59,851 cases and 113,154 controls. SNPs absent in the outcome dataset were excluded, and we did not use proxy SNPs. We harmonised exposure and outcome datasets using the ‘harmonise_data’ function in ‘Two-Sample MR’ package (version 0.5.11; University of Bristol, Bristol, UK) to align allele orientation and exclude ambiguous variants. A total of 29 palindromic SNPs with intermediate allele frequencies (between 0.4 and 0.6) were excluded to ensure accurate strand alignment [17].

### Statistical Analysis

We applied five two-sample MR techniques to estimate the causal effect of VAT on MDD: weighted median (WM), inverse-variance weighted (IVW), MR-Egger regression, simple mode and weighted mode. The IVW method combined SNP-specific Wald ratios using a meta-analytic approach under the assumption that all IVs are valid [21]. To evaluate the robustness of our findings, we conducted several sensitivity analyses, including MR-Egger intercept testing, Cochran’s Q test, funnel plot asymmetry, Mendelian randomisation Pleiotropy RESidual Sum and Outlier (MR-PRESSO) and leave-one-out analysis.

Heterogeneity across SNP-specific estimates was assessed using Cochran’s Q test and the I^2^ statistic, with I^2^ >25% and *p* < 0.05 indicating significant heterogeneity [22]. MR-Egger intercept was used to detect horizontal pleiotropy [22], and funnel plot symmetry was visually inspected to assess potential directional bias. Leave-one-out sensitivity analysis plot was performed to examine the influence of individual SNPs on the overall causal estimate. Additionally, MR-PRESSO was applied to detect and correct for outlier SNPs, which were removed and re-analysed to obtain corrected causal estimates [23].

All analyses were performed using R (version 4.3.3; R Foundation for Statistical Computing, Vienna, Austria) with the Two-Sample MR package (version 0.5.11; University of Bristol, Bristol, UK) and MR-PRESSO (version 1.0; McGill University, Montreal, Canada) [17]. MR estimates were expressed as odds ratios (ORs) with 95% confidence intervals (CIs), calculated via exponential transformation of the MR coefficients. An association was considered statistically significant if *p* < 0.05.

## Results

### Genetic IV Screening

Of the 221 VAT-associated SNPs, three SNPs (rs112108364, rs34431565 and rs9277979) absent from the MDD dataset were excluded from analysis. Throughout the harmonisation procedure, 29 SNPs (rs10423928, rs10740991, rs11161044, rs117176448, rs12001634, rs12335914, rs145350287, rs1454687, rs148168215, rs2253310, rs2537621, rs2730806, rs3787075, rs3791687, rs3943933, rs4419475, rs4562625, rs61537964, rs62477685, rs67463976, rs7021721, rs71658797, rs73213484, rs754635, rs7654647, rs7942037, rs8074454, rs8103728 and rs9304665) were not retained for analysis due to palindromic alignment issues related to moderate allele frequencies. Identified as outliers through MR-PRESSO, the SNPs rs254024 and rs2804477 were eliminated. Ultimately, a re-analysis was carried out utilising the remaining 187 SNPs as IVs to derive revised causal estimates.

### Causal Effect of VAT on MDD

**Fig. 2.**
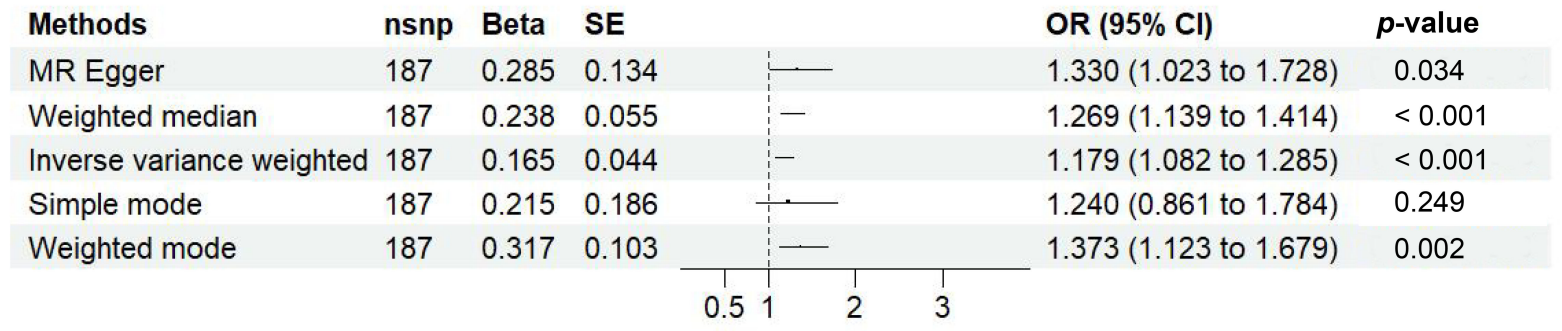
**MR findings regarding the connection between VAT and MDD**. *p*-values below 0.05 reflected a causal relationship between VAT and MDD. VAT, visceral adipose tissue; MDD, major depressive disorder; nsnp, number of single nucleotide polymorphisms; Beta, regression coefficient; SE, standard error; OR, odds ratio; CI, confidence interval.

**Fig. 3.**
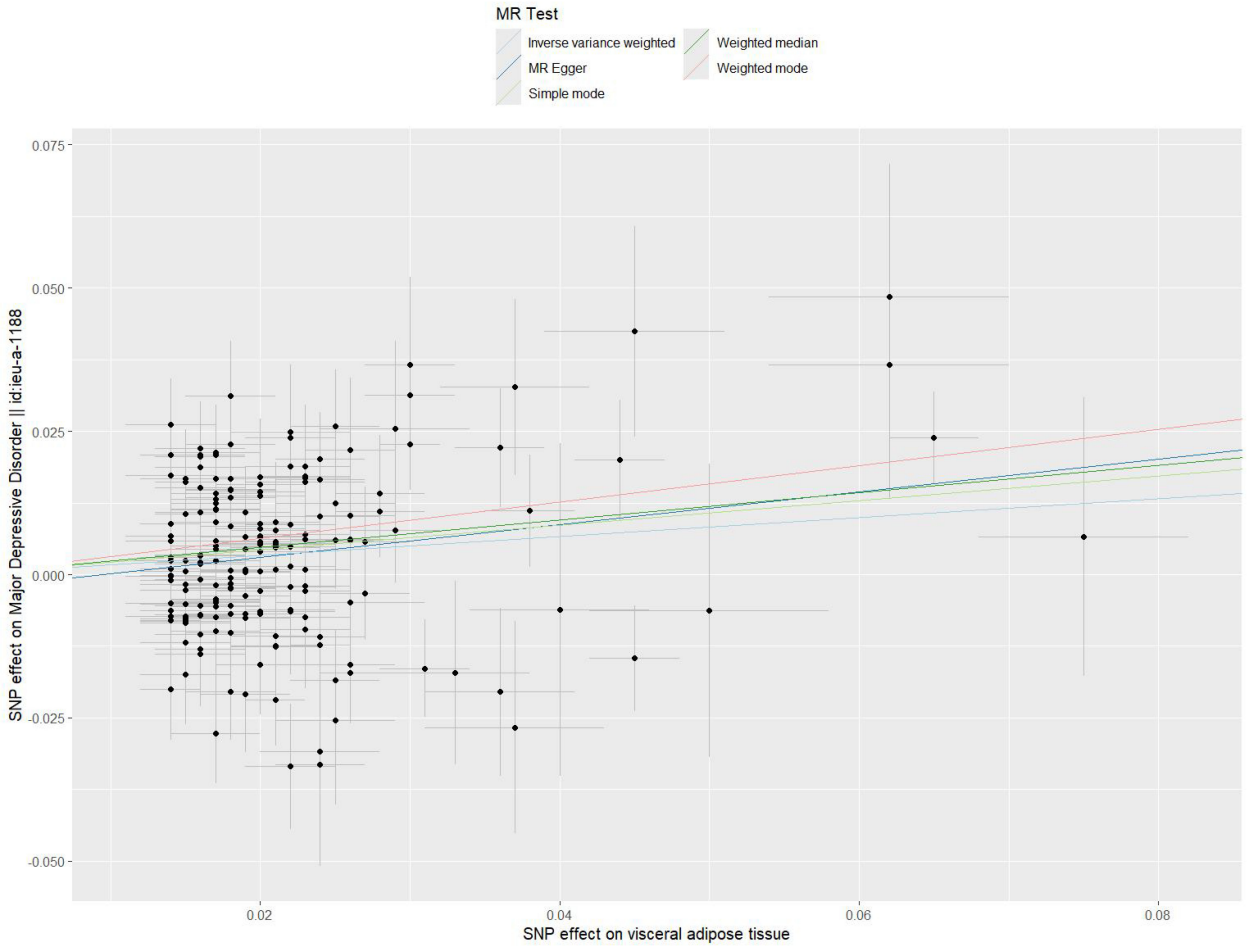
**Scatter plot of SNP-specific effects for VAT on MDD**. Each point represents one of the 187 SNPs, with the x-axis showing SNP effects on VAT and the y-axis showing corresponding effects on MDD. Regression lines represent different MR methods, including IVW, WM, MR–Egger, weighted mode and simple mode. VAT, visceral adipose tissue; MDD, major depressive disorder; SNP, single nucleotide polymorphism; MR, Mendelian randomisation; IVW, inverse-variance weighted; WM, weighted median.

Fig. 2 illustrates the results of MR analysis. Our results revealed that each standard deviation increase in genetically predicted VAT was associated with a 17.9% elevated risk of MDD (OR = 1.179, 95% CI: 1.082–1.285, *p* < 0.001), with consistent effect directions and statistical significance confirmed by the MR Egger, WM and weighted mode methods. Collectively, these results indicated that an elevated VAT mass was related to an increased risk of MDD. Fig. 3 displays the scatter plot illustrating the MR analysis between VAT and MDD.

### Sensitivity Analyses

**Fig. 4.**
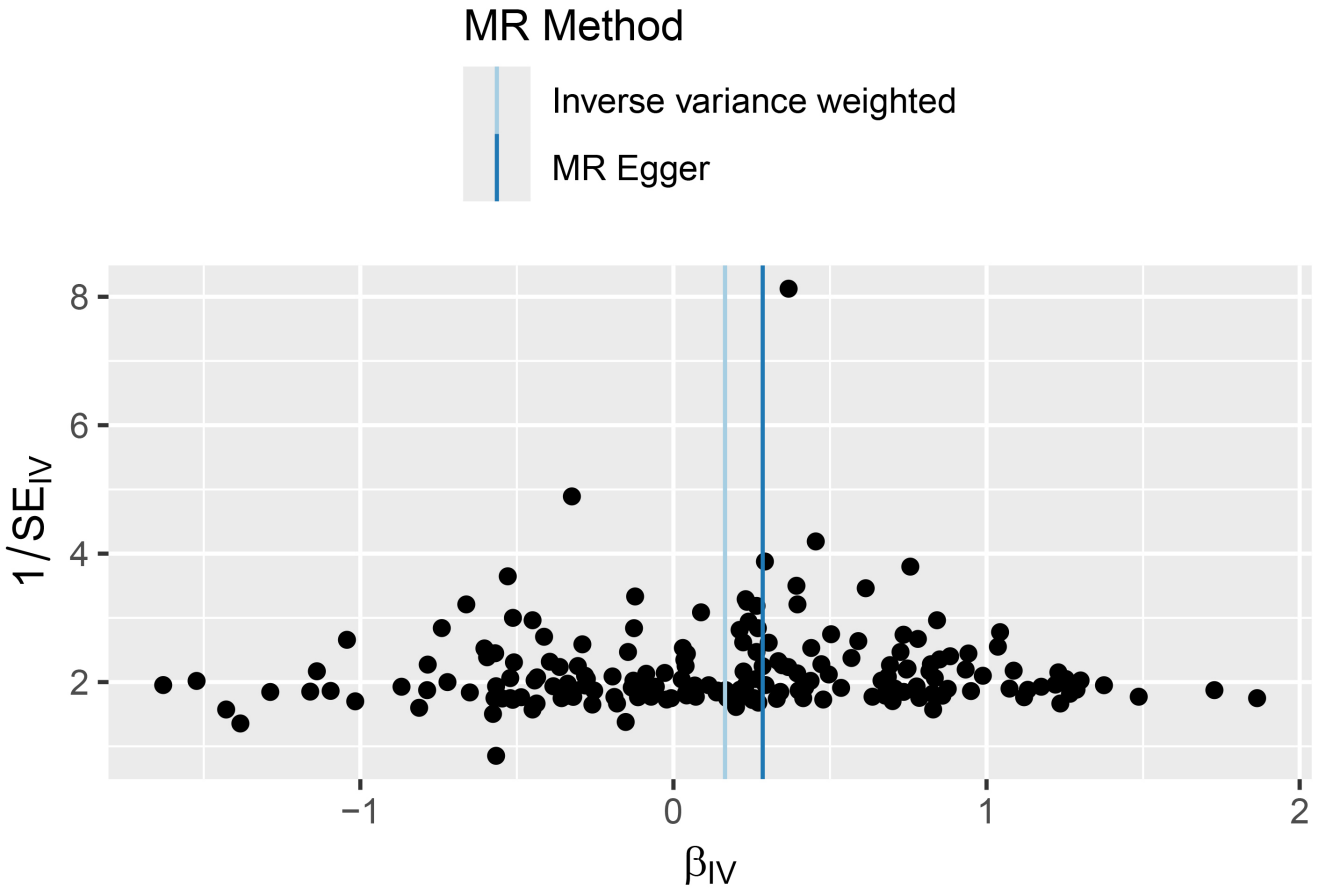
**Funnel plot depicting the effect of VAT on MDD**. MR, mendelian randomisation; VAT, visceral adipose tissue; MDD, major depressive disorder.

**Fig. 5.**
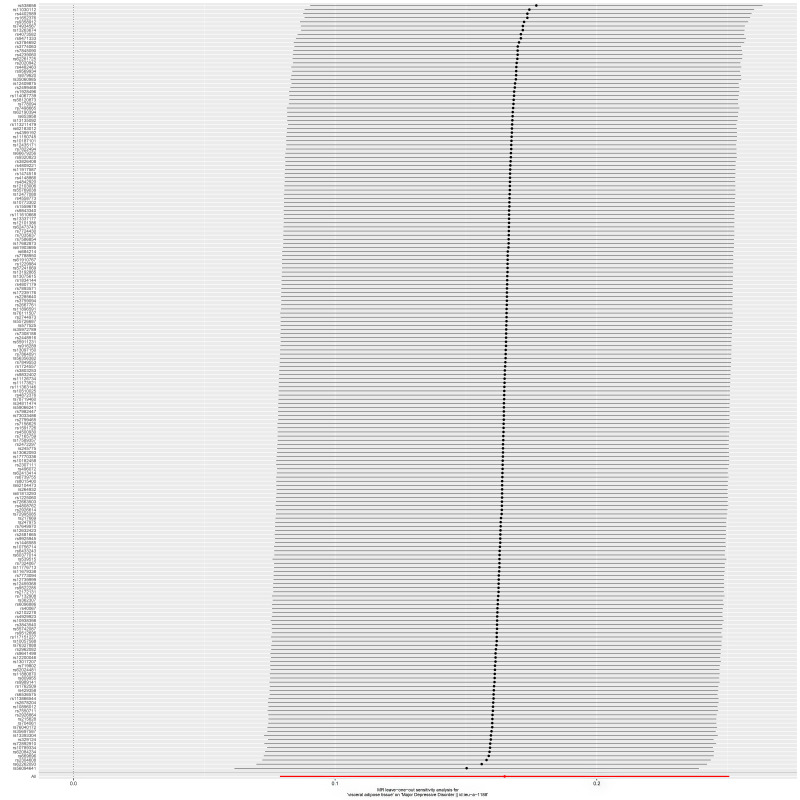
**Leave-one-out test of the causal effect of VAT on MDD**. Each point represents the causal estimate obtained after removing one SNP at a time from the set of 187 VAT-associated variants. The x-axis shows the estimated OR and 95% CI for MDD risk. The red dashed line indicates the overall MR estimate. The results demonstrated that no single SNP had a significant influence on the overall effect estimate. VAT, visceral adipose tissue; MDD, major depressive disorder; SNP, single nucleotide polymorphism; MR, Mendelian randomisation.

Sensitivity analyses consistently supported the robustness of the causal relationship between VAT and MDD. Significant heterogeneity was detected (Cochran’s Q = 351.415, *p* < 0.001 for MR Egger; Q = 353.140, *p* < 0.001 for IVW; Table 1), but the random-effects IVW method provided stable estimates under heterogeneity. The MR–Egger intercept indicated no evidence of directional pleiotropy (intercept = –0.003, *p* = 0.342; Table 2), and funnel plots showed symmetrical distribution of SNP effects, further suggesting the absence of unbalanced pleiotropy (Fig. 4). Leave-one-out analysis demonstrated that no single SNP significantly influenced the overall estimate (Fig. 5).

**Table 1.** **Heterogeneity analysis in 187 VAT genetic variants from the MDD GWAS data set**.

Method	Q_value	Q_df	*p*-value
MR Egger	351.415	185	<0.001
Inverse variance weighted	353.140	186	<0.001

Abbreviations: MR, mendelian randomisation; VAT, visceral adipose tissue; MDD, major depressive disorder; GWAS, genome-wide association study.

**Table 2.** **Pleiotropy testing for 198 VAT variants in the MDD GWAS dataset**.

GWAS dataset	Egger_intercept	se	*p*-value
MDD	−0.003	0.003	0.342

Abbreviations: VAT, visceral adipose tissue; MDD, major depressive disorder; GWAS, genome-wide association study; se, standard error.

MR-PRESSO identified two outlier SNPs (rs254024 and rs2804477), which were excluded from the final analysis. After outlier correction, the causal association remained unchanged (OR = 1.176, 95% CI: 1.079–1.282, *p* < 0.001), reinforcing the robustness of the findings.

Together, these results strengthen the validity of the VAT–MDD causal relationship.

## Discussion

In this study, we applied a two-sample MR approach using genome-wide significant SNPs associated with VAT to explore its causal effect on MDD. Our findings demonstrated that a genetically predicted increase in VAT mass was causally associated with an elevated risk of MDD, with each standard deviation increase in VAT corresponding to an estimated 18% rise in risk. Prior studies have suggested that VAT may serve as a marker of metabolic dysfunction; however, our MR analysis specifically supported a potential causal relationship between VAT and MDD risk [16]. These results suggested that VAT may contribute to the development of depressive disorders.

Although numerous observational studies have reported a correlation between VAT and MDD, they have been limited by residual confounding and reverse causation [24, 25]. Our MR analysis addressed these limitations by utilising genetic variants as IVs, thereby enabling a reliable estimation of causality. This finding was aligned with mechanistic evidence that VAT functions as an endocrine organ capable of secreting pro-inflammatory cytokines like IL-1β and TNF-α, which cross the blood–brain barrier to induce neuroinflammation, particularly in the hippocampus, contributing to depressive symptoms [7, 9]. For instance, experimental models have shown that VAT accumulation in mice—resulting from either high-fat diet or sub-chronic social stress—correlates with hypothalamic macrophage infiltration and hepatic metabolic disturbances, which may contribute to neuroinflammatory processes relevant to MDD pathophysiology, although depressive-like behaviors were not directly assessed in these studies [26, 27].

In addition to inflammatory pathways, VAT may influence the risk of MDD via neuroendocrine and psychosocial mechanisms. VAT accumulation has been linked to HPA axis dysregulation and elevated cortisol levels, which are commonly observed in individuals with MDD and may exacerbate depressive symptoms through chronic stress signalling [28, 29, 30]. Moreover, VAT-associated adipokine imbalances, such as leptin resistance and reduced adiponectin, have been linked to altered mood regulation [31, 32]. Psychosocial stress and stigma associated with visceral obesity may exacerbate these biological effects, creating a feedback loop that reinforces depressive symptoms [33].

Notably, our results remained consistent across multiple MR sensitivity analyses, including MR–Egger, WM and MR-PRESSO. Although significant heterogeneity was detected (Cochran’s Q *p* < 0.001), this does not necessarily indicate invalid instruments, as it may reflect polygenic architecture, population-specific LD structures, or unaccounted gene–environment interactions. The MR-Egger intercept test showed no evidence of directional pleiotropy, and leave-one-out analysis confirmed that no single SNP significantly influenced the causal estimate [34]. Furthermore, MR-PRESSO identified and corrected two outlier SNPs, after which the association remained unchanged, reinforcing the robustness of our findings [35].

Despite these strengths, several limitations should be acknowledged. Firstly, our study population was limited to individuals of European ancestry due to the available GWAS datasets. Consequently, the generalisability of our findings to non-European populations remains uncertain. Future studies should replicate these findings in diverse cohorts, including individuals of Asian, African and Latin American descent, to assess population-specific effects. Secondly, our design reduced confounding, but we could not eliminate the possibility that some VAT-associated SNPs may also influence MDD through related pathways, such as overall obesity (e.g., BMI). Although we selected VAT-specific SNPs and conducted multiple pleiotropy tests, future studies should apply multivariable MR frameworks that include BMI and other adiposity traits to elucidate shared genetic effects. Additionally, we were unable to conduct sex-stratified analyses because of the lack of gender-specific outcome data, which may be important given known sex differences in fat distribution and MDD prevalence.

In summary, this study provides genetic evidence supporting a causal relationship between VAT and the development of MDD. These findings suggested that VAT is a modifiable risk factor and potential therapeutic target for preventing or mitigating MDD. Future longitudinal and interventional studies, as well as multivariable MR analyses in diverse populations, are warranted to validate and expand these findings.

## Conclusion

This research demonstrated a causal relationship between VAT mass and MDD. Our findings suggested that VAT served as a marker of metabolic dysfunction and a modifiable risk factor for depression. Targeted interventions to reduce VAT, such as lifestyle modifications or anti-inflammatory therapies, may offer promising strategies for the prevention and management of MDD. These results highlight the potential of incorporating VAT assessment into predictive models for MDD and warrant further investigation in diverse populations and clinical settings.

## Availability of Data and Materials

The data used in this study were publicly available. Summary data of VAT can be obtained from the GWAS Catalog: https://www.ebi.ac.uk/gwas/downloads/summary-statistics. The MDD GWAS datasets can be obtained from the IEU OpenGWAS project at https://gwas.mrcieu.ac.uk/.

## Supplementary Material

Supplementary material associated with this article can be found, in the online version, at https://doi.org/10.62641/aep.v53i5.1972.
