# Supplementary material for: Effect of Visceral Adipose Tissue on Major Depressive Disorder: A Mendelian Randomisation Research
Source: Actas Esp Psiquiatr. 2025 Oct 5;53(5):1083–92. doi: 10.62641/aep.v53i5.1972 (PMC12538619; doi:10.62641/aep.v53i5.1972)
Supplement: Supplementary file 1 [file ActEsp-53-5-1083-1092-s1.docx]

**Supplementary table 1** Information on instrumental variables from the VAT dataset

| **SNP** | **EA** | **OA** | **EAF** | **BETA** | **SE** | **P** | **R^2^** | **F** |
| --- | --- | --- | --- | --- | --- | --- | --- | --- |
| rs56094641 | G | A | 0.771 | 0.065 | 0.003 | < 0.001 | 2.02E-03 | 658.12 |
| rs538656 | T | G | 0.724 | 0.045 | 0.003 | < 0.001 | 7.42E-04 | 241.56 |
| rs13393304 | A | G | 0.877 | -0.044 | 0.003 | < 0.001 | 5.66E-04 | 184.11 |
| rs539515 | C | A | 0.805 | 0.038 | 0.003 | < 0.001 | 4.76E-04 | 154.95 |
| rs62262093 | T | C | 0.365 | -0.030 | 0.002 | < 0.001 | 4.49E-04 | 145.93 |
| rs11030112 | A | G | 0.756 | 0.031 | 0.003 | < 0.001 | 4.30E-04 | 140.00 |
| rs76111507 | T | C | 0.989 | -0.075 | 0.007 | < 0.001 | 4.05E-04 | 131.72 |
| rs10938398 | A | G | 0.675 | 0.028 | 0.003 | < 0.001 | 3.94E-04 | 128.22 |
| rs72892910 | T | G | 0.820 | 0.036 | 0.003 | < 0.001 | 3.68E-04 | 119.73 |
| rs7498665 | G | A | 0.739 | 0.027 | 0.003 | < 0.001 | 3.48E-04 | 113.32 |
| rs4808762 | C | T | 0.791 | 0.029 | 0.003 | < 0.001 | 3.44E-04 | 112.01 |
| rs10182458 | G | A | 0.441 | 0.026 | 0.002 | < 0.001 | 3.41E-04 | 111.00 |
| rs4402589 | T | G | 0.454 | -0.026 | 0.002 | < 0.001 | 3.41E-04 | 110.92 |
| rs10423928 | A | T | 0.828 | -0.033 | 0.003 | < 0.001 | 3.38E-04 | 109.93 |
| rs2307111 | C | T | 0.384 | -0.026 | 0.003 | < 0.001 | 3.24E-04 | 105.25 |
| rs7132908 | A | G | 0.748 | 0.026 | 0.003 | < 0.001 | 3.09E-04 | 100.49 |
| rs9471333 | C | T | 0.548 | 0.024 | 0.002 | < 0.001 | 2.96E-04 | 96.16 |
| rs113211479 | A | G | 0.664 | 0.024 | 0.003 | < 0.001 | 2.88E-04 | 93.69 |
| rs3784692 | C | T | 0.463 | -0.024 | 0.003 | < 0.001 | 2.85E-04 | 92.83 |
| rs669696 | A | C | 0.714 | -0.024 | 0.003 | < 0.001 | 2.85E-04 | 92.68 |
| rs17770336 | T | C | 0.790 | 0.025 | 0.003 | < 0.001 | 2.69E-04 | 87.64 |
| rs10740991 | G | C | 0.854 | 0.026 | 0.003 | < 0.001 | 2.66E-04 | 86.60 |
| rs1454687 | C | G | 0.453 | 0.023 | 0.002 | < 0.001 | 2.66E-04 | 86.38 |
| rs71658797 | A | T | 0.971 | 0.035 | 0.004 | < 0.001 | 2.58E-04 | 84.06 |
| rs9320823 | T | C | 0.689 | -0.023 | 0.003 | < 0.001 | 2.58E-04 | 84.01 |
| rs7156625 | A | G | 0.805 | 0.027 | 0.003 | < 0.001 | 2.50E-04 | 81.25 |
| rs6096886 | G | A | 0.827 | -0.028 | 0.003 | < 0.001 | 2.49E-04 | 80.92 |
| rs6739755 | A | G | 0.708 | 0.023 | 0.003 | < 0.001 | 2.46E-04 | 79.89 |
| rs429358 | C | T | 0.849 | -0.030 | 0.003 | < 0.001 | 2.45E-04 | 79.61 |
| rs2678204 | G | T | 0.745 | 0.023 | 0.003 | < 0.001 | 2.39E-04 | 77.76 |
| rs9358912 | T | G | 0.634 | -0.025 | 0.003 | < 0.001 | 2.39E-04 | 77.67 |
| rs2304608 | A | C | 0.701 | 0.030 | 0.003 | < 0.001 | 2.36E-04 | 76.78 |
| rs35060985 | A | G | 0.701 | 0.023 | 0.003 | < 0.001 | 2.34E-04 | 76.23 |
| rs1591726 | T | C | 0.589 | 0.022 | 0.003 | < 0.001 | 2.18E-04 | 70.75 |
| rs1652376 | T | G | 0.390 | -0.021 | 0.002 | < 0.001 | 2.17E-04 | 70.70 |
| rs1225060 | A | G | 0.835 | 0.023 | 0.003 | < 0.001 | 2.14E-04 | 69.44 |
| rs62190394 | T | C | 0.724 | 0.022 | 0.003 | < 0.001 | 2.08E-04 | 67.59 |
| rs76040172 | A | G | 0.935 | -0.045 | 0.006 | < 0.001 | 2.06E-04 | 67.08 |
| rs2253310 | C | G | 0.470 | -0.021 | 0.003 | < 0.001 | 2.05E-04 | 66.70 |
| rs62084234 | G | A | 0.703 | 0.025 | 0.003 | < 0.001 | 2.04E-04 | 66.40 |
| rs117151227 | C | T | 0.977 | -0.062 | 0.008 | < 0.001 | 2.02E-04 | 65.82 |
| rs62261725 | G | A | 0.711 | -0.021 | 0.003 | < 0.001 | 2.02E-04 | 65.60 |
| rs12477088 | C | T | 0.533 | -0.020 | 0.003 | < 0.001 | 2.01E-04 | 65.38 |
| rs2285640 | G | A | 0.592 | 0.020 | 0.002 | < 0.001 | 2.00E-04 | 64.97 |
| rs4239060 | A | G | 0.865 | -0.026 | 0.003 | < 0.001 | 1.99E-04 | 64.64 |
| rs879620 | C | T | 0.344 | -0.021 | 0.003 | < 0.001 | 1.99E-04 | 64.63 |
| rs2744973 | T | C | 0.591 | 0.021 | 0.003 | < 0.001 | 1.94E-04 | 63.05 |
| rs10756714 | G | A | 0.622 | -0.020 | 0.002 | < 0.001 | 1.91E-04 | 62.05 |
| rs7550711 | T | C | 0.988 | 0.062 | 0.008 | < 0.001 | 1.88E-04 | 61.18 |
| rs9989141 | C | T | 0.506 | -0.020 | 0.003 | < 0.001 | 1.87E-04 | 60.66 |
| rs62477685 | T | A | 0.554 | -0.019 | 0.003 | < 0.001 | 1.86E-04 | 60.39 |
| rs13017207 | A | G | 0.716 | -0.020 | 0.003 | < 0.001 | 1.85E-04 | 60.07 |
| rs13062093 | G | T | 0.632 | 0.020 | 0.003 | < 0.001 | 1.82E-04 | 59.09 |
| rs4482463 | C | A | 0.778 | 0.036 | 0.005 | < 0.001 | 1.81E-04 | 58.88 |
| rs8015400 | C | A | 0.553 | -0.020 | 0.003 | < 0.001 | 1.74E-04 | 56.65 |
| rs11880870 | G | A | 0.321 | -0.018 | 0.002 | < 0.001 | 1.72E-04 | 55.78 |
| rs55726687 | A | G | 0.848 | 0.023 | 0.003 | < 0.001 | 1.72E-04 | 55.78 |
| rs1928496 | C | T | 0.746 | -0.021 | 0.003 | < 0.001 | 1.71E-04 | 55.51 |
| rs10896012 | C | T | 0.863 | 0.022 | 0.003 | < 0.001 | 1.71E-04 | 55.48 |
| rs9522285 | A | G | 0.716 | 0.019 | 0.003 | < 0.001 | 1.70E-04 | 55.44 |
| rs113866544 | C | T | 0.926 | 0.037 | 0.005 | < 0.001 | 1.69E-04 | 55.01 |
| rs66679256 | T | C | 0.556 | 0.018 | 0.002 | < 0.001 | 1.69E-04 | 54.87 |
| rs40067 | A | G | 0.738 | -0.024 | 0.003 | < 0.001 | 1.67E-04 | 54.46 |
| rs3843540 | C | T | 0.598 | -0.026 | 0.003 | < 0.001 | 1.66E-04 | 53.85 |
| rs11150745 | G | A | 0.790 | -0.019 | 0.003 | < 0.001 | 1.63E-04 | 53.14 |
| rs7845090 | G | A | 0.641 | 0.020 | 0.003 | < 0.001 | 1.63E-04 | 53.09 |
| rs653958 | G | A | 0.684 | 0.019 | 0.003 | < 0.001 | 1.63E-04 | 53.03 |
| rs62104473 | T | C | 0.832 | 0.019 | 0.003 | < 0.001 | 1.63E-04 | 52.90 |
| rs55742087 | T | C | 0.847 | -0.023 | 0.003 | < 0.001 | 1.62E-04 | 52.84 |
| rs13135092 | G | A | 0.975 | 0.033 | 0.005 | < 0.001 | 1.58E-04 | 51.46 |
| rs2481665 | C | T | 0.813 | -0.018 | 0.002 | < 0.001 | 1.58E-04 | 51.33 |
| rs9277979 | T | C | 0.882 | 0.023 | 0.003 | < 0.001 | 1.57E-04 | 51.1 |
| rs12459368 | G | A | 0.694 | -0.020 | 0.003 | < 0.001 | 1.56E-04 | 50.58 |
| rs9641499 | A | C | 0.603 | -0.018 | 0.003 | < 0.001 | 1.55E-04 | 50.49 |
| rs577525 | T | C | 0.403 | -0.018 | 0.002 | < 0.001 | 1.54E-04 | 50.02 |
| rs72663503 | T | C | 0.882 | 0.021 | 0.003 | < 0.001 | 1.53E-04 | 49.86 |
| rs1834144 | A | C | 0.555 | -0.018 | 0.003 | < 0.001 | 1.53E-04 | 49.78 |
| rs13337177 | T | G | 0.791 | -0.023 | 0.003 | < 0.001 | 1.52E-04 | 49.47 |
| rs245775 | A | G | 0.759 | -0.020 | 0.003 | < 0.001 | 1.52E-04 | 49.33 |
| rs76327888 | T | G | 0.710 | 0.023 | 0.003 | < 0.001 | 1.50E-04 | 48.75 |
| rs4929923 | T | C | 0.533 | -0.018 | 0.003 | < 0.001 | 1.48E-04 | 48.24 |
| rs2926614 | T | C | 0.764 | -0.022 | 0.003 | < 0.001 | 1.48E-04 | 48.05 |
| rs56356382 | C | T | 0.795 | -0.022 | 0.003 | < 0.001 | 1.45E-04 | 47.17 |
| rs61910767 | T | C | 0.946 | -0.023 | 0.003 | < 0.001 | 1.45E-04 | 47.04 |
| rs719802 | T | C | 0.493 | 0.017 | 0.003 | < 0.001 | 1.44E-04 | 46.81 |
| rs7893571 | G | T | 0.796 | -0.018 | 0.003 | < 0.001 | 1.42E-04 | 46.03 |
| rs4073582 | A | G | 0.834 | -0.018 | 0.003 | < 0.001 | 1.41E-04 | 45.97 |
| rs3787075 | G | C | 0.695 | 0.018 | 0.003 | < 0.001 | 1.41E-04 | 45.87 |
| rs35697587 | G | A | 0.407 | 0.017 | 0.002 | < 0.001 | 1.40E-04 | 45.39 |
| rs7982447 | C | T | 0.702 | 0.021 | 0.003 | < 0.001 | 1.40E-04 | 45.38 |
| rs7649970 | T | C | 0.880 | 0.025 | 0.004 | < 0.001 | 1.39E-04 | 45.20 |
| rs2172131 | T | C | 0.706 | 0.017 | 0.003 | < 0.001 | 1.39E-04 | 45.12 |
| rs145350287 | A | T | 0.990 | -0.042 | 0.006 | < 0.001 | 1.39E-04 | 45.06 |
| rs61813293 | T | G | 0.953 | 0.024 | 0.004 | < 0.001 | 1.39E-04 | 45.04 |
| rs3943933 | A | T | 0.427 | 0.017 | 0.002 | < 0.001 | 1.38E-04 | 44.80 |
| rs4558773 | A | G | 0.716 | 0.017 | 0.003 | < 0.001 | 1.38E-04 | 44.75 |
| rs2926864 | A | G | 0.813 | 0.017 | 0.003 | < 0.001 | 1.37E-04 | 44.40 |
| rs10187101 | T | C | 0.712 | -0.017 | 0.003 | < 0.001 | 1.36E-04 | 44.39 |
| rs111610668 | G | A | 0.852 | -0.017 | 0.003 | < 0.001 | 1.36E-04 | 44.19 |
| rs12103006 | A | G | 0.533 | -0.017 | 0.003 | < 0.001 | 1.35E-04 | 44.02 |
| rs2730806 | T | A | 0.439 | 0.016 | 0.002 | < 0.001 | 1.35E-04 | 43.96 |
| rs7035637 | A | G | 0.667 | 0.019 | 0.003 | < 0.001 | 1.35E-04 | 43.84 |
| rs8074454 | C | G | 0.706 | 0.017 | 0.003 | < 0.001 | 1.35E-04 | 43.74 |
| rs72995085 | C | T | 0.909 | -0.021 | 0.003 | < 0.001 | 1.34E-04 | 43.73 |
| rs704061 | C | T | 0.501 | 0.016 | 0.002 | < 0.001 | 1.34E-04 | 43.64 |
| rs11679338 | C | T | 0.716 | -0.017 | 0.003 | < 0.001 | 1.34E-04 | 43.52 |
| rs12739999 | A | G | 0.616 | 0.022 | 0.003 | < 0.001 | 1.32E-04 | 43.00 |
| rs7942037 | C | G | 0.711 | -0.017 | 0.003 | < 0.001 | 1.32E-04 | 42.94 |
| rs7308188 | C | T | 0.554 | -0.019 | 0.003 | < 0.001 | 1.32E-04 | 42.92 |
| rs4500930 | T | C | 0.632 | 0.017 | 0.003 | < 0.001 | 1.32E-04 | 42.9 |
| rs7822494 | C | T | 0.549 | -0.016 | 0.002 | < 0.001 | 1.31E-04 | 42.65 |
| rs34811474 | A | G | 0.926 | -0.019 | 0.003 | < 0.001 | 1.31E-04 | 42.53 |
| rs684214 | T | C | 0.770 | 0.018 | 0.003 | < 0.001 | 1.30E-04 | 42.17 |
| rs13192865 | A | G | 0.813 | -0.018 | 0.003 | < 0.001 | 1.29E-04 | 42.08 |
| rs67463976 | C | G | 0.511 | 0.016 | 0.003 | < 0.001 | 1.29E-04 | 42.00 |
| rs7165759 | A | G | 0.728 | -0.017 | 0.003 | < 0.001 | 1.28E-04 | 41.78 |
| rs74934567 | G | A | 0.882 | -0.022 | 0.003 | < 0.001 | 1.28E-04 | 41.54 |
| rs12335914 | C | G | 0.616 | 0.016 | 0.002 | < 0.001 | 1.27E-04 | 41.44 |
| rs3791687 | T | A | 0.692 | 0.019 | 0.003 | < 0.001 | 1.27E-04 | 41.40 |
| rs114067739 | A | C | 0.975 | -0.037 | 0.006 | < 0.001 | 1.26E-04 | 40.84 |
| rs9843340 | C | T | 0.931 | -0.022 | 0.003 | < 0.001 | 1.25E-04 | 40.74 |
| rs112108364 | G | T | 0.860 | 0.017 | 0.003 | < 0.001 | 1.21E-04 | 39.51 |
| rs217669 | C | T | 0.642 | 0.017 | 0.003 | < 0.001 | 1.21E-04 | 39.48 |
| rs55769038 | G | A | 0.372 | -0.016 | 0.003 | < 0.001 | 1.21E-04 | 39.44 |
| rs13075615 | T | C | 0.931 | -0.022 | 0.003 | < 0.001 | 1.20E-04 | 39.13 |
| rs254024 | T | G | 0.583 | 0.016 | 0.002 | < 0.001 | 1.20E-04 | 39.02 |
| rs10789334 | A | G | 0.915 | -0.018 | 0.003 | < 0.001 | 1.20E-04 | 39.00 |
| rs6433243 | T | C | 0.649 | 0.016 | 0.003 | < 0.001 | 1.19E-04 | 38.86 |
| rs13263674 | G | A | 0.861 | 0.017 | 0.003 | < 0.001 | 1.19E-04 | 38.82 |
| rs215628 | C | T | 0.398 | 0.016 | 0.003 | < 0.001 | 1.17E-04 | 38.17 |
| rs1474518 | C | T | 0.761 | -0.018 | 0.003 | < 0.001 | 1.17E-04 | 38.13 |
| rs2962082 | A | G | 0.614 | -0.015 | 0.002 | < 0.001 | 1.17E-04 | 38.10 |
| rs7654647 | T | A | 0.700 | 0.016 | 0.003 | < 0.001 | 1.17E-04 | 38.06 |
| rs1724557 | C | A | 0.372 | 0.016 | 0.003 | < 0.001 | 1.17E-04 | 38.06 |
| rs9925945 | C | A | 0.782 | -0.017 | 0.003 | < 0.001 | 1.17E-04 | 38.01 |
| rs2667761 | C | T | 0.492 | -0.016 | 0.003 | < 0.001 | 1.17E-04 | 37.95 |
| rs809955 | A | G | 0.646 | -0.016 | 0.003 | < 0.001 | 1.17E-04 | 37.90 |
| rs10773302 | G | T | 0.803 | -0.017 | 0.003 | < 0.001 | 1.16E-04 | 37.85 |
| rs12001634 | A | T | 0.531 | -0.016 | 0.003 | < 0.001 | 1.16E-04 | 37.79 |
| rs11126734 | A | C | 0.654 | -0.015 | 0.002 | < 0.001 | 1.16E-04 | 37.77 |
| rs35972789 | A | C | 0.990 | -0.040 | 0.006 | < 0.001 | 1.15E-04 | 37.30 |
| rs11776713 | C | T | 0.626 | -0.015 | 0.002 | < 0.001 | 1.15E-04 | 37.25 |
| rs4148866 | T | C | 0.603 | 0.015 | 0.003 | < 0.001 | 1.13E-04 | 36.84 |
| rs4399192 | G | T | 0.726 | 0.018 | 0.003 | < 0.001 | 1.11E-04 | 36.17 |
| rs2499468 | C | A | 0.775 | -0.016 | 0.003 | < 0.001 | 1.11E-04 | 36.00 |
| rs13097150 | T | C | 0.699 | 0.015 | 0.003 | < 0.001 | 1.11E-04 | 35.94 |
| rs362307 | T | C | 0.972 | 0.029 | 0.005 | < 0.001 | 1.10E-04 | 35.93 |
| rs1762509 | A | G | 0.775 | 0.016 | 0.003 | < 0.001 | 1.10E-04 | 35.79 |
| rs2020942 | T | C | 0.745 | 0.015 | 0.003 | < 0.001 | 1.10E-04 | 35.72 |
| rs3803253 | A | G | 0.797 | -0.016 | 0.003 | < 0.001 | 1.10E-04 | 35.62 |
| rs778094 | G | A | 0.342 | 0.015 | 0.003 | < 0.001 | 1.09E-04 | 35.48 |
| rs78719460 | A | G | 0.864 | 0.016 | 0.003 | < 0.001 | 1.09E-04 | 35.29 |
| rs1229984 | T | C | 0.841 | -0.050 | 0.008 | < 0.001 | 1.09E-04 | 35.29 |
| rs62183012 | C | T | 0.879 | -0.016 | 0.003 | < 0.001 | 1.08E-04 | 35.20 |
| rs12435171 | G | A | 0.388 | 0.016 | 0.003 | < 0.001 | 1.08E-04 | 35.19 |
| rs9832402 | G | A | 0.685 | -0.017 | 0.003 | < 0.001 | 1.08E-04 | 35.19 |
| rs61903695 | G | A | 0.854 | 0.017 | 0.003 | < 0.001 | 1.08E-04 | 35.16 |
| rs2472297 | T | C | 0.934 | 0.017 | 0.003 | < 0.001 | 1.08E-04 | 35.13 |
| rs754635 | C | G | 0.766 | -0.023 | 0.004 | < 0.001 | 1.08E-04 | 35.01 |
| rs2102278 | G | A | 0.455 | 0.016 | 0.003 | < 0.001 | 1.07E-04 | 34.90 |
| rs117176448 | G | C | 0.963 | 0.025 | 0.004 | < 0.001 | 1.07E-04 | 34.79 |
| rs4562625 | C | G | 0.666 | 0.015 | 0.003 | < 0.001 | 1.07E-04 | 34.74 |
| rs9569934 | T | C | 0.773 | -0.019 | 0.003 | < 0.001 | 1.06E-04 | 34.61 |
| rs73213484 | T | A | 0.839 | -0.021 | 0.004 | < 0.001 | 1.06E-04 | 34.56 |
| rs1559678 | C | T | 0.441 | 0.015 | 0.003 | < 0.001 | 1.06E-04 | 34.52 |
| rs55911231 | T | C | 0.559 | 0.015 | 0.003 | < 0.001 | 1.06E-04 | 34.51 |
| rs60377014 | T | C | 0.882 | -0.020 | 0.003 | < 0.001 | 1.06E-04 | 34.38 |
| rs2804477 | A | G | 0.849 | 0.021 | 0.004 | < 0.001 | 1.05E-04 | 34.00 |
| rs7586854 | T | C | 0.398 | -0.014 | 0.002 | < 0.001 | 1.04E-04 | 33.97 |
| rs12200046 | T | C | 0.932 | 0.022 | 0.004 | < 0.001 | 1.04E-04 | 33.87 |
| rs2799465 | C | T | 0.711 | 0.021 | 0.004 | < 0.001 | 1.04E-04 | 33.83 |
| rs3774063 | T | C | 0.959 | 0.024 | 0.004 | < 0.001 | 1.03E-04 | 33.64 |
| rs62473743 | A | G | 0.734 | 0.020 | 0.003 | < 0.001 | 1.03E-04 | 33.59 |
| rs3759094 | T | C | 0.733 | -0.015 | 0.003 | < 0.001 | 1.03E-04 | 33.52 |
| rs12409875 | A | G | 0.644 | -0.014 | 0.002 | < 0.001 | 1.03E-04 | 33.48 |
| rs247975 | T | C | 0.631 | -0.014 | 0.002 | < 0.001 | 1.03E-04 | 33.41 |
| rs3826408 | T | C | 0.613 | 0.014 | 0.002 | < 0.001 | 1.02E-04 | 33.29 |
| rs8103728 | C | G | 0.618 | -0.015 | 0.003 | < 0.001 | 1.02E-04 | 33.27 |
| rs61537964 | G | C | 0.857 | -0.023 | 0.004 | < 0.001 | 1.02E-04 | 33.25 |
| rs62413414 | T | C | 0.927 | 0.020 | 0.003 | < 0.001 | 1.02E-04 | 33.09 |
| rs11917587 | A | G | 0.484 | 0.014 | 0.003 | < 0.001 | 1.02E-04 | 33.06 |
| rs34431565 | T | G | 0.967 | -0.034 | 0.006 | < 0.001 | 1.02E-04 | 33.02 |
| rs17589357 | C | T | 0.937 | -0.020 | 0.003 | < 0.001 | 1.01E-04 | 32.72 |
| rs62024481 | T | C | 0.845 | -0.018 | 0.003 | < 0.001 | 1.00E-04 | 32.60 |
| rs1446585 | G | A | 0.190 | -0.017 | 0.003 | < 0.001 | 9.99E-05 | 32.48 |
| rs7773094 | C | T | 0.738 | -0.018 | 0.003 | < 0.001 | 9.99E-05 | 32.48 |
| rs7324067 | T | C | 0.775 | -0.017 | 0.003 | < 0.001 | 9.97E-05 | 32.42 |
| rs2537621 | C | G | 0.676 | 0.015 | 0.003 | < 0.001 | 9.95E-05 | 32.34 |
| rs10510025 | T | C | 0.634 | 0.016 | 0.003 | < 0.001 | 9.92E-05 | 32.25 |
| rs7849553 | C | A | 0.593 | 0.014 | 0.002 | < 0.001 | 9.89E-05 | 32.16 |
| rs4807179 | G | A | 0.394 | -0.015 | 0.003 | < 0.001 | 9.88E-05 | 32.12 |
| rs6536575 | T | C | 0.494 | -0.014 | 0.002 | < 0.001 | 9.86E-05 | 32.08 |
| rs148168215 | T | A | 0.984 | -0.053 | 0.009 | < 0.001 | 9.84E-05 | 32.01 |
| rs9304665 | T | A | 0.521 | -0.017 | 0.003 | < 0.001 | 9.78E-05 | 31.82 |
| rs7021721 | C | G | 0.723 | -0.015 | 0.003 | < 0.001 | 9.77E-05 | 31.77 |
| rs11173521 | T | G | 0.398 | 0.014 | 0.003 | < 0.001 | 9.71E-05 | 31.57 |
| rs12632423 | A | G | 0.840 | -0.023 | 0.004 | < 0.001 | 9.64E-05 | 31.36 |
| rs4842920 | T | G | 0.882 | -0.015 | 0.003 | < 0.001 | 9.58E-05 | 31.16 |
| rs7724430 | A | C | 0.470 | 0.014 | 0.003 | < 0.001 | 9.52E-05 | 30.95 |
| rs4872376 | C | T | 0.552 | -0.014 | 0.002 | < 0.001 | 9.51E-05 | 30.93 |
| rs58120873 | A | G | 0.938 | -0.025 | 0.004 | < 0.001 | 9.51E-05 | 30.93 |
| rs17239176 | C | T | 0.896 | -0.017 | 0.003 | < 0.001 | 9.47E-05 | 30.81 |
| rs7864091 | A | G | 0.828 | 0.019 | 0.003 | < 0.001 | 9.46E-05 | 30.76 |
| rs73033486 | A | G | 0.905 | 0.021 | 0.004 | < 0.001 | 9.45E-05 | 30.73 |
| rs17682873 | T | C | 0.924 | 0.020 | 0.004 | < 0.001 | 9.43E-05 | 30.66 |
| rs4809221 | G | A | 0.652 | -0.015 | 0.003 | < 0.001 | 9.41E-05 | 30.62 |
| rs916289 | T | C | 0.714 | -0.014 | 0.002 | < 0.001 | 9.39E-05 | 30.55 |
| rs496072 | T | C | 0.295 | 0.014 | 0.003 | < 0.001 | 9.38E-05 | 30.50 |
| rs2448916 | A | C | 0.472 | -0.014 | 0.003 | < 0.001 | 9.34E-05 | 30.36 |
| rs111363146 | C | T | 0.933 | 0.020 | 0.004 | < 0.001 | 9.33E-05 | 30.33 |
| rs11896591 | G | A | 0.638 | 0.014 | 0.002 | < 0.001 | 9.33E-05 | 30.33 |
| rs264932 | A | G | 0.825 | 0.014 | 0.003 | < 0.001 | 9.32E-05 | 30.31 |
| rs4419475 | T | A | 0.418 | 0.014 | 0.003 | < 0.001 | 9.32E-05 | 30.30 |
| rs10057588 | G | A | 0.692 | -0.015 | 0.003 | < 0.001 | 9.30E-05 | 30.23 |
| rs57241669 | G | A | 0.812 | -0.026 | 0.005 | < 0.001 | 9.28E-05 | 30.18 |
| rs9512696 | A | G | 0.471 | -0.014 | 0.003 | < 0.001 | 9.28E-05 | 30.16 |
| rs329124 | G | A | 0.538 | -0.014 | 0.003 | < 0.001 | 9.24E-05 | 30.04 |
| rs59066241 | G | T | 0.767 | 0.021 | 0.004 | < 0.001 | 9.21E-05 | 29.95 |
| rs11161044 | G | C | 0.659 | -0.017 | 0.003 | < 0.001 | 9.20E-05 | 29.91 |
| rs12101386 | T | G | 0.858 | -0.016 | 0.003 | < 0.001 | 9.14E-05 | 29.72 |
| rs7788950 | A | G | 0.811 | -0.017 | 0.003 | < 0.001 | 9.14E-05 | 29.72 |

Abbreviations: VAT, visceral adipose tissue, SNP, single nucleotide polymorphism; EA, Effect allele; OA, Other allele; SE, standard error.
